# Unmasking the Mind: A Journey Through Misdiagnosis to the True Identity of Dissociative Identity Disorder

**DOI:** 10.1192/bjo.2025.10706

**Published:** 2025-06-20

**Authors:** Nadia Amira Ashikin

**Affiliations:** Hospital Putrajaya, WP Putrajaya, Malaysia

## Abstract

**Aims:** Dissociative Identity Disorder (DID) is a complex psychiatric condition that is often misdiagnosed due to its overlapping symptoms with other disorders such as mood and psychotic disorders. The presence of psychotic features, including auditory and visual hallucinations, disorganized behaviour, and memory gaps, can make the diagnosis of DID particularly challenging. This case study highlights a 27-year-old female whose DID diagnosis was delayed due to misinterpretation of her psychotic symptoms, which were initially attributed to other psychiatric disorders.

**Methods:** A 27-year-old female with a 15-year history of psychiatric care began experiencing symptoms at the age of 13, initially presenting with anxiety and panic attacks. Over time, her symptoms escalated to include episodes of auditory and visual hallucinations, disorganized speech, and erratic behaviour, leading to multiple hospitalizations. During one hospitalization, she displayed regressive behaviours, mutism, aggressive outbursts, hypomania, and dissociative amnesia. Despite extensive workups, including MRI scans and lab tests, no organic causes were found. Her diagnosis fluctuated between psychotic disorders, mood disorders, anxiety disorder, and dissociative disorder. Her mood and psychotic symptoms were initially treated as schizoaffective disorder, but the patient experienced adverse reactions to antipsychotic medications, including galactorrhoea from risperidone and weight gain from amisulpride. These medications were ineffective, prompting a reassessment of her diagnosis. A thorough review of her clinical history, including reports of memory gaps, identity disturbances, and dissociative episodes, led to the reconsideration of DID as the primary diagnosis.

**Results:** The psychotic features in this patient, such as hallucinations and disorganized behaviour, were secondary to her dissociative episodes, occurring during times of identity disturbance. This case underscores that psychotic symptoms in DID can easily be misinterpreted as part of a mood or psychotic disorder, especially when dissociative episodes are not initially recognized. The prolonged misdiagnosis delayed appropriate treatment, but a more comprehensive understanding of her symptoms led to the correct diagnosis and tailored management.

**Conclusion:** This case highlights the diagnostic challenges in identifying DID, particularly when psychotic features overlap with other psychiatric conditions. Early recognition of DID, with a thorough longitudinal assessment of both dissociative and psychotic symptoms, is crucial for accurate diagnosis and improving patient outcomes. A more targeted approach, including trauma-informed care and psychotherapy, would have been beneficial and should be considered in similar cases.

